# Noise-Resistant Demosaicing with Deep Image Prior Network and Random RGBW Color Filter Array

**DOI:** 10.3390/s22051767

**Published:** 2022-02-24

**Authors:** Edwin Kurniawan, Yunjin Park, Sukho Lee

**Affiliations:** 1 Department of Computer Engineering, Dongseo University, Busan 47011, Korea; edwin.kurniawan.93@gmail.com; 2 Department of Mathematics, Ewha University, Seoul 03760, Korea; yunjjiin@naver.com

**Keywords:** color filter array, deep image prior, demosaicing, deep learning

## Abstract

In this paper, we propose a deep-image-prior-based demosaicing method for a random RGBW color filter array (CFA). The color reconstruction from the random RGBW CFA is performed by the deep image prior network, which uses only the RGBW CFA image as the training data. To our knowledge, this work is a first attempt to reconstruct the color image with a neural network using only a single RGBW CFA in the training. Due to the White pixels in the RGBW CFA, more light is transmitted through the CFA than in the case with the conventional RGB CFA. As the image sensor can detect more light, the signal-to-noise-ratio (SNR) increases and the proposed demosaicing method can reconstruct the color image with a higher visual quality than other existing demosaicking methods, especially in the presence of noise. We propose a loss function that can train the deep image prior (DIP) network to reconstruct the colors from the White pixels as well as from the red, green, and blue pixels in the RGBW CFA. Apart from using the DIP network, no additional complex reconstruction algorithms are required for the demosaicing. The proposed demosaicing method becomes useful in situations when the noise becomes a major problem, for example, in low light conditions. Experimental results show the validity of the proposed method for joint demosaicing and denoising.

## 1. Introduction

Current digital imaging systems often consist of monochromatic image sensors equipped with color filter arrays to capture color information. Color filters are required because the photosensors response to the intensity of the light and cannot distinguish the color information. Therefore, a small filter is coated in front of each pixel so that the small filter filters the light by wavelength range to obtain information about the specific color of light for each pixel. For example, the Bayer CFA pattern [1] passes through only one of the primary colors (Red, Green and Blue) at each pixel, where the ratio of the numbers of the R, G, and B pixels in this pattern is 1:2:1. The other two missing colors have to be reconstructed by demosaicing algorithms [2,3,4,5,6,7,8,9,10,11].

However, the problem of low resolution arises with the Bayer CFA because information about two color components is lost for each pixel. Moreover, as much of the light is absorbed by the color filters, the proportion of the light energy to the noise energy decreases, which again results in a decrease of the signal-to-noise-ratio (SNR). Therefore, the reconstructed (demosaiced) color image becomes noisy, especially, in low illumination environments when the light energy is low. The problem of noise intensifies as the resolution of mobile photos in smartphones grows since the small sized sensors receive less light and which causes a loss of detail, resulting in relatively high noise levels. The noise problem deepens as the resolution of mobile photos in smartphones increases, reducing the size of image sensors and the light energy they detect. Therefore, to allow more of the incident light to be detected rather than absorbed, CFAs using secondary colors (Cyan, Yellow, and Magenta) have been proposed in various forms, including CYGM (Cyan, Yellow, Green, Magenta), the CMY (Cyan, Magenta, and Yellow), and the CMYW (Cyan, Magenta, Yellow, and White) CFAs [12,13]. Other demosaicing methods use the RGBW (Red, Green, Blue, and White) CFA, which is a CFA that contains ‘White’ pixels that allow for the penetration of all color intensities [14,15]. There is a tradeoff between the number of R, G, and B pixels and the number of W pixels in demosaicing methods using the RGBW CFA as a higher number of W pixels results in a larger sensitivity of the W sensor, which makes it more robust to the noise, but this also results in a degradation of the resolution as white pixels do not contain color information. In this paper, we propose a deep image prior (DIP)-based demosaicing method that can reconstruct colors from a random RGBW CFA without the use of any complex reconstruction algorithm other than the training of the DIP with the single CFA image. To train the DIP network, we propose a new loss function that is tailored for the demosaicing of the random RGBW CFA. The loss function differs from existing ones used in DIP-based image restoration problems in the respect that for the white pixels, it imposes a linear constraint that the generated color components should obey instead of specifying the values of the color components. This is the first time that the DIP is used for the demosaicing of the CFA, which contains white pixels.

## 2. Related Works

### 2.1. Color Filter Arrays in Digital Imaging Systems

**Figure 1:**
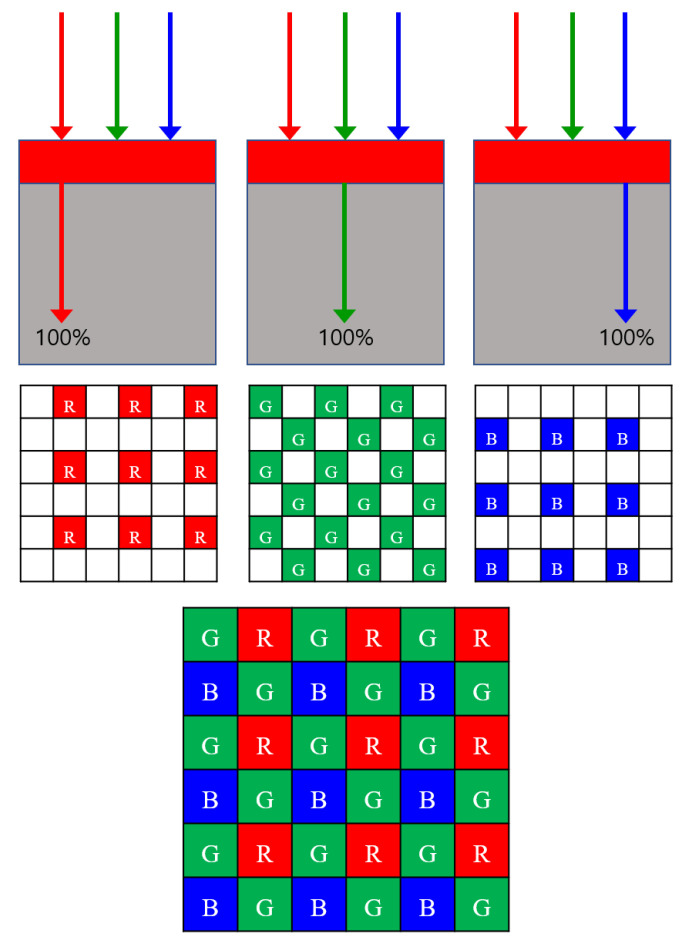
A 6×6 partial cut of the Bayer color filter array. R: Red pixels, G: Green pixels, B: Blue pixels.

Current digital imaging systems often constitute monochrome image sensors that are overlaid with color filter arrays(CFAs) to capture color information. The most commonly used CFA is the Bayer CFA, which consists of Red, Green, and Blue color filters so that only one color component can be obtained at each pixel location, as shown in Figure 1. Let Ω be the two-dimensional spatial domain of the image, $I_{orig}\left[k\right]={\left[R_{orig}\left[k\right],G_{orig}\left[k\right],B_{orig}\left[k\right]\right]}^{T}$ be the true color image triple at k, and $c\left[k\right]={\left[c_{R}\left[k\right],c_{G}\left[k\right],c_{B}\left[k\right]\right]}^{T}$ be the action of the CFA. Then, the intensity value $I_{s}\left[k\right]$ of the Red (or Green/Blue) component sensed at the position k∈Ω in the Bayer CFA can be expressed as the inner product of $I_{orig}\left[k\right]$ and c[k]:

(1) $$I_{s}\left[k\right]=c{\left[k\right]}^{T}I_{orig}\left[k\right].$$

For the Bayer CFA, the components of c[k] are defined as

(2) $$\begin{array}{cc} c_{R}\left[k\right]=1,c_{G}\left[k\right]=0,c_{B}\left[k\right]=0 & \mathrm{if}\;k\in S_{R}\\ c_{R}\left[k\right]=0,c_{G}\left[k\right]=1,c_{B}\left[k\right]=0 & \mathrm{if}\;k\in S_{G}\\ c_{R}\left[k\right]=0,c_{G}\left[k\right]=0,c_{B}\left[k\right]=1 & \mathrm{if}\;k\in S_{B} \end{array}$$

where $S_{R}$, $S_{G}$, and $S_{B}$ denote the sets of the R, G, and B pixels, respectively. The problem of demosaicing is to restore $I_{orig}$ from $I_{s}$, which is an ill-posed problem since $I_{orig}$ is a three-channel image, while the sensed image $I_{s}$ is a one-channel monochrome image. Therefore, additional constraints have to be imposed to solve the demosaicing problem, such as applying an additional assumption that adjacent pixels have similar colors. By applying this assumption, in conventional demosaicing methods, the missing two color components are interpolated from the spatially adjacent CFA data.

### 2.2. RGBW Color Filter Array

In [16], the proportions of the R, G, and B components in the white pixel are computed based on the assumption that a linear relationship exists between the white component and the three primary color components (red, green, and blue). Based on this assumption, the components in RGBW CFA matrices, which contain R, G, B, and W (white) pixels, can be defined as:

(3) $$\begin{array}{cc} c_{R}\left[k\right]=1,c_{G}\left[k\right]=0,c_{B}\left[k\right]=0 & \mathrm{if}\;k\in S_{R}\\ c_{R}\left[k\right]=0,c_{G}\left[k\right]=1,c_{B}\left[k\right]=0 & \mathrm{if}\;k\in S_{G}\\ c_{R}\left[k\right]=0,c_{G}\left[k\right]=0,c_{B}\left[k\right]=1 & \mathrm{if}\;k\in S_{B}\\ c_{R}\left[k\right]=\alpha_{R},c_{G}\left[k\right]=\alpha_{G},c_{B}\left[k\right]=\alpha_{B} & \mathrm{if}\;k\in S_{W} \end{array}$$

where $S_{W}$ denotes the set of the white pixels, and $\alpha_{R}$, $\alpha_{G}$, and $\alpha_{B}$ are the proportions of the R, G, and B components, respectively, in the white pixel. The values for $\alpha_{R}$, $\alpha_{G}$, and $\alpha_{B}$ differ for different sensors. For example, in [16], the authors calculated $\alpha_{R}$, $\alpha_{G}$, and $\alpha_{B}$ by optimization to obtain $\alpha_{R}=0.2936$, $\alpha_{G}=0.4905$, and $\alpha_{B}=0.2159$ using a typical RGBW sensor, i.e., the VEML6040 sensor developed by the Vishay company [17]. In this paper, we use the same values, $\alpha_{R}$, $\alpha_{G}$, and $\alpha_{B}$, as calculated in [16] but emphasize the fact that the proposed method works with any values of $\alpha_{R}$, $\alpha_{G}$, and $\alpha_{B}$.

It can be seen from (3) that the demosaicing problem can no longer be solved by mere interpolation as with the Bayer CFA because the intensity values sensed at the W pixels become linear combinations of the R, G, and B color components. Therefore, as a set of linear equations have to be solved, more complex demosaicing methods have to be applied, such as those in [12,13] in conventional non-neural network approaches. Meanwhile, neural network approaches for demosaicing use an end-to-end approach where the input is the mosaiced image and the output the ground-truth image. Therefore, the loss function is simple where the output of the network is to be compared with the ground-truth image. For example, the work of [18] uses the loss function defined as

(4) $$L=\sum_{i}{∥N_{\theta }\left(I_{i}^{in}\right)-I_{i}^{t}∥}^{2},$$

where $N_{\theta }\left(\cdot \right)$ denotes the neural network, and $I_{i}^{in}$ and $I_{i}^{t}$ are the *i*’s mosaiced and ground-truth images, respectively. Other tasks, such as the work of [19,20] we used in the comparison in the experiments, divide the entire image into smaller patches but apply the same loss function as shown in (4) to all the patches:

(5) $$L=\sum_{i}\sum_{p_{i}^{k}}{∥N_{\theta }\left(I_{p_{i}^{k}}^{in}\right)-I_{p_{i}^{k}}^{t}∥}^{2},$$

However, conventional neural network approaches require a large dataset of mosaic/ground-truth images for the training of the neural network, and the training process usually takes several days. In comparison, we propose the use of a deep image prior (DIP) network that can be trained on the single sensed CFA image and can simultaneously obtain the color components for the R, G, B, and W pixels based on a loss function tailored for this problem.

### 2.3. Deep-Image-Prior-Based Image Restoration

Recently, Ulyanov et al. proposed the use of the DIP for image restoration [21]. The DIP resembles an auto encoder but is trained with a single image I, i.e., the image to be restored. The DIP converts a noise tensor z derived from a uniform distribution into a restored image $g_{\theta }\left(z\right)$, which is the output of the deep image prior network with parameters θ and input z. In [21], the DIP is applied to the task of inpainting by minimizing the following loss function:

(6) $$L=∥\left(g_{\theta }\left(z\right)-I\right){⊙m∥}^{2},$$

where ⊙ is the Hadamard’s product(element-wise multiplication operator) and $m\in {\left\{0,1\right\}}^{H\times W}$ is a binary mask with a value equal to zero corresponding to the missing pixels to be inpainted and a value of one corresponding to the pixels to be preserved. It has been shown in [21] that the minimization of L in (6) with respect to θ results in a DIP network that can inpaint an image. Inpainting and demosaicing are similar in that they fill in the missing information but differ in the fact that in inpainting the existing pixels have full channel information while in demosaicing they have not. That is, while all R, G, and B values are available for existing pixels in inpainting, in demosaicing, existing pixels have only one of the R, G, and B values. In [22], Park et al. use the variational DIP for the joint task of demosaicing and denoising. However, so far the DIP has never been used for the demosaicing of CFA images which contain white pixels.

## 3. Proposed Method

In this section, we explain in detail the proposed method. We first introduce the random RGBW color filter array. Then, we propose a method how to reconstruct the colors from the monochrome CFA image sensed with the random RGBW CFA.

### 3.1. Random RGBW Color Filter Array

In neural network-based demosaicing methods, it is the CFA that determines the parameters of the neural network because the CFA defines the loss function. A different CFA results in a different loss function, which means that the resulting parameters of the network become different for different CFAs. The reason that we come up with a random pattern is based on the guess that teaching a network to generate random colors at random locations would make it easy to find the most meaningful parameters for the backpropagation because there is no bias for specific colors at certain locations. Furthermore, the reason that we used equal numbers of R, G, and B pixels instead of the widely used ratio of 1:2:1 for R, G, and B pixels is that in the reconstructed image, the color seed should serve as a reference point for the R, G, and B colors of pixels around the color seed to revive. When the number of R, G, and B color seeds is unbalanced, then the color will revive itself disproportionately, as we will show in the experimental section.

**Figure 2:**
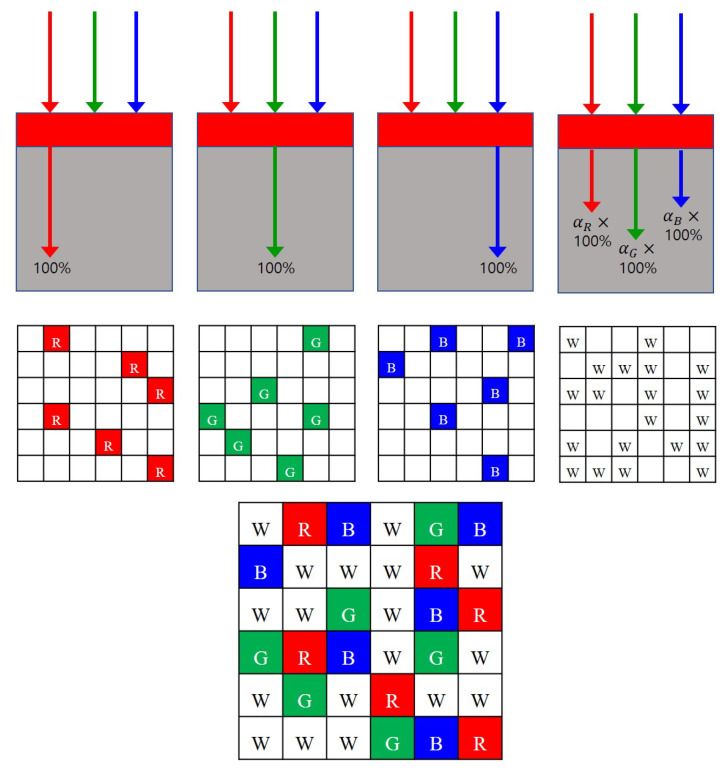
Proposed random RGBW CFA. R: Red pixels, G: Green pixels, B: Blue pixels, W: White pixels.

Figure 2 shows a 6×6 partial cut of the proposed random RGBW CFA pattern. Fifty percent of the whole RGBW CFA consists of white pixels, and the remaining 50% is divided equally between R, G and B pixels. The value of the sensed intensity value $I_{s}\left[k\right]$ contains, according to (1), components of $c_{R}\left[k\right],c_{G}\left[k\right],c_{B}\left[k\right]$ defined as in (3). The components of $c_{R}\left[k\right],c_{G}\left[k\right],c_{B}\left[k\right]$ are constrained to lie in [0, 1] for physical feasibility since they correspond to the opacity rates. It is known that the white pixels let more of the light energy through than the R, G, and B pixels. As more light energy is detected at the white pixels, the signal-to-noise ratio is larger than at the R, G, and B pixels. This makes the random RGBW CFA more robust against the noise.

### 3.2. DIP-Based Demosaicing of the Random RGBW-CFA

In this section, we propose a DIP-based demosaicing method for the demosaicing of the random RGBW CFA. We denote by $g_{\theta }\left(z\right)$ the three-dimensional output of the DIP with network parameters θ and z as the input. The size of $g_{\theta }\left(z\right)$ is H×W×3, where *H* and *W* are the height and the width of the demosaiced image, respectively, and the number of channels is 3, corresponding to the R, G, and B channels. At every pixel k∈Ω, where Ω is the two-dimensional spatial domain of $g_{\theta }\left(z\right)$, the color is defined by an RGB triplet $g_{\theta }\left(z\right)\left[k\right]$, which is a three-element vector that specifies the intensities of the Red, Green, and Blue components of the color. For the purpose of simplification, we will denote $g_{\theta }\left(z\right)\left[k\right]$ as $g_{\theta }\left[k\right]$. Thus,

(7) $$g_{\theta }\left(z\right)\left[k\right]=g_{\theta }\left[k\right]={\left[g_{\theta }^{R}\left[k\right],g_{\theta }^{G}\left[k\right],g_{\theta }^{B}\left[k\right]\right]}^{T},$$

where $g_{\theta }^{R}\left[k\right]$, $g_{\theta }^{G}\left[k\right]$, and $g_{\theta }^{B}\left[k\right]$ are the Red, Green, and Blue components of the three-element vector $g_{\theta }\left(z\right)\left[k\right]$. The loss function used in the training of the DIP is

(8) $$L=\sum_{k\in \Omega }{∥c{\left[k\right]}^{T}g_{\theta }\left[k\right]-I_{s}\left[k\right]∥}^{2}$$

where the components of $c\left[k\right]={\left[c_{R}\left[k\right],c_{G}\left[k\right],c_{B}\left[k\right]\right]}^{T}$ are defined as in (3). The minimization of the loss function constrains the output $g_{\theta }\left[k\right]$ to obey the physical constraint imposed on the sensed monochrome CFA image, i.e., the constraint that the sensed monochrome pixel is a weighted sum of the R, G, and B components, where the weights are determined by the ratio of opacity of the color filters in the CFA. It should be noticed that the form of the loss function is different from those designed for other image restoration purposes with the DIP. In conventional DIPs, pixel-wise comparisons are made between the intensities of the generated pixels and the data pixels. In comparison, the loss function in (8) does not make a pixel-wise comparison between the intensities but imposes a constraint that the generated pixel values should obey. The reason that the original colors can be restored by minimizing the loss function in (8) lies in the fact that the components of c[k] vary for neighboring pixels and that the DIP works as a prior that favors a natural image. If the vectors c[k] are the same for all k, numerous images of different colors will be exist that minimize the loss in (8), making it difficult to restore the original colors. However, as the vectors c[k] are different for neighboring pixels, there exists a unique solution that satisfies both the constraint of making the loss function in (8) minimum and the image prior constraint imposed by the DIP. However, even though there exists a unique solution, it is not guaranteed that the DIP can find this solution. The R (or G, B) pixels act as anchor points that hold the true R (or G, B) color components of the sensed image. As the other components of c[k] at these pixels are zero, the true R color components will be reconstructed using these pixels when minimizing the loss in (8). The other color components will use these reconstructed true color components as clues and will be reconstructed by satisfying the constraints imposed by the loss in (8) and the DIP.

Writing the loss function in (8) in matrix form results in

(9) $$L=∥\hat{RGB2Gray}\left(c⊙g_{\theta }\left(z\right)\right)-I_{s}{∥}^{2}$$

where ⊙ denotes the element-wise product and $\hat{RGB2Gray}\left(\cdot \right)$ denotes the operation similar to the RGB to Gray image conversion, i.e., the operation that produces a monochrome image by adding the R, G, and B components. Here, the operation of $\hat{RGB2Gray}\left(\cdot \right)$ is adding the three components in the three channels of $c⊙g_{\theta }\left(z\right)$, resulting in a one-channel image. It should be noted that for the R, G, and B pixels, i.e., for $k\in S_{R}\cup S_{G}\cup S_{B}$ (where ∪ denotes the union operator), only one component in $c\left[k\right]⊙g_{\theta }\left(z\right)\left[k\right]$ is non-zero as c[k] has only one non-zero component. This is why we use the notation $\hat{RGB2Gray}\left(\cdot \right)$ instead of RGB2Gray(·).

However, if the loss function in (9) is minimized directly, the colors in the demosaiced image will fade. This is due to the fact that the minimizing of the loss function in (9) reaches the local minimum without sufficiently reconstructing the color components inherent in the white pixels. To prevent this phenomenon, we split up the loss function in (9) into four terms and apply different weights to them:

(10) $$L=\lambda_{W}L_{W}+\lambda_{R}L_{R}+\lambda_{G}L_{G}+\lambda_{B}L_{B}.$$

Here, $L_{W}$ is related to the white pixels, and $L_{R}$, $L_{G}$, and $L_{B}$ are related to the R, G, and B pixels, respectively, where $L_{W}$ is defined as

(11) $$L_{W}={∥M_{W}⊙\left[\hat{RGB2Gray}\left(c⊙g_{\theta }\left(z\right)\right)-I_{s}\right]∥}^{2}$$

and

(12) $$L_{R}={∥M_{R}⊙\left[\hat{RGB2Gray}\left(c⊙g_{\theta }\left(z\right)\right)-I_{s}\right]∥}^{2},$$

(13) $$L_{G}={∥M_{G}⊙\left[\hat{RGB2Gray}\left(c⊙g_{\theta }\left(z\right)\right)-I_{s}\right]∥}^{2},$$

(14) $$L_{B}={∥M_{B}⊙\left[\hat{RGB2Gray}\left(c⊙g_{\theta }\left(z\right)\right)-I_{s}\right]∥}^{2}.$$

Here, the mask $M_{W}$ has values of 1 at the white pixel positions and 0 at the other positions. Similarly, $M_{R}$ ($M_{G}$,$M_{B}$) has a value of 1 at the Red (Green, Blue) pixel positions and 0 at the other pixel positions. The sizes of $M_{W}$, $M_{R}$, $M_{G}$, and $M_{B}$ are all H×W.

If $\lambda_{W}=\lambda_{R}=\lambda_{G}=\lambda_{B}=1$, the losses in (9) and (10) are the same. To avoid the color fading artifact, we apply different values of $\lambda_{W},\lambda_{R},\lambda_{G}$ and $\lambda_{B}$ for different iteration number *t*:

(15) $$\begin{array}{ccccc} \lambda_{W}=1, & \lambda_{R}=1, & \lambda_{G}=0, & \lambda_{B}=0 & t<500\\ \lambda_{W}=1, & \lambda_{R}=0, & \lambda_{G}=1, & \lambda_{B}=0 & 500\leq t<1000\\ \lambda_{W}=1, & \lambda_{R}=0, & \lambda_{G}=0, & \lambda_{B}=1 & 1000\leq t<1500\\ \lambda_{W}=1.5, & \lambda_{R}=0.5, & \lambda_{G}=0.5, & \lambda_{B}=0.5 & 1500\leq t\leq 2100 \end{array}$$

For the first 500 iterations, the color image is reconstructed using only the R pixels and the White pixels, i.e., by minimizing only $\lambda_{W}L_{W}+\lambda_{R}L_{R}$ with $\lambda_{W}=\lambda_{R}=1$. By doing so, we expect that the red colors are fully reconstructed at least at the R pixels. After that, we reconstruct the colors for the next 500 iterations according to the sensed G pixels and White pixels, i.e., $\lambda_{W}L_{W}+\lambda_{G}L_{G}$ with $\lambda_{W}=\lambda_{G}=1$, and then, for the next 500 iterations, according to the sensed B pixels and White pixels, i.e., $\lambda_{W}L_{W}+\lambda_{B}L_{B}$ with $\lambda_{W}=\lambda_{B}=1$. At the end of 1500 iterations, the R, G, and B color components are highly saturated at all pixels. We then run another 600 iterations with $\lambda_{W}=1.5,\;\lambda_{R}=0.5,\;\lambda_{G}=0.5$, and $\lambda_{B}=0.5$. This imposes a strong linear constraint on the white pixels according to the minimization of the loss function in (11) while also trying to maintain the reconstructed colors to some extent. The number of iterations has been determined by experiments.

**Figure 3:**
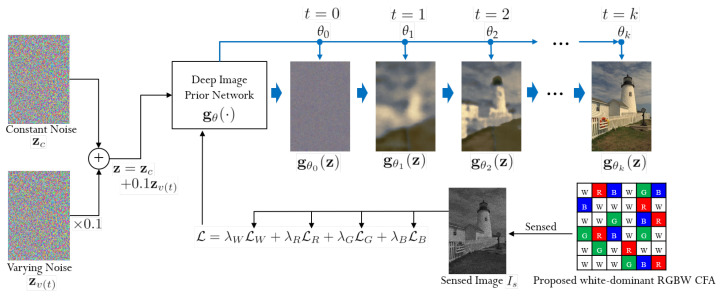
Overall diagram of the proposed method.

Figure 3 shows the overall diagram of the proposed method and how the input noise is gradually turning into the demosaiced image as the optimization process progresses according to the minimization of the loss function in (8). We use a noise image z as the input to the DIP, which is sum of a constant noise ($z_{c}$) and a variable noise ($z_{v(t)}$), as in the variational DIP in [22]. The variable noise $z_{v(t)}$ is newly generated for each step of the training and is multiplied by 0.1 before being added to $z_{c}$:

(16) $$z=z_{c}+0.1z_{v(t)}.$$

The constant noise $z_{c}$ remains unchanged throughout the training. After the training of the DIP is finished, i.e., at the test time, only the constant noise $z_{c}$ is put into the DIP to obtain the final denoised and demosaiced color image. The reason that $z_{v(t)}$ is added to $z_{c}$ in the training is to obtain a denoising effect, as explained in [22], i.e., the extra variable noise $z_{v(t)}$ prevents the output of the DIP from being noisy.

## 4. Experiments and Discussion

### 4.1. Experimental Settings

We experimented on the noise-added Kodak [23] dataset, which contains 24 images in bmp image file format with size of 768×512, and the McMaster dataset, which is used in the experiments in [24] consisting of 18 color images with resolution of 500×500. The noise is derived from a zero-mean Gaussian distribution with different standard deviations for each color channel. We set the standard deviations as 0.0463, 0.0294, 0.0322, and 0.0157 for the R, G, B, and W channels, respectively. The standard deviations are determined according to actual physical measurements of the amount of noises in the R, G, B, and W channels acquired under low light condition. The amount of noise is different for each channel since each color filter absorbs different light energy.

### 4.2. Network Structure

We use the same structure of the DIP network as in [21] for inpainting. The network has an encoder-decoder type structure that consists of five down-sampling and five up-sampling convolutional layers with skip connections, where each convolutional layer consists of 128 feature maps obtained by 3×3 convolutions followed by a Leaky ReLU activation. The weights in the DIP are all initialized with a Gaussian noise with standard deviation of 0.03. For the backpropagation optimization, we used the Adam optimizer with a learning rate of 0.001.

### 4.3. Experimental Results

We compared the proposed method with other joint demosaicing and denoising neural networks such as the Sequential Energy Minimization (SEM) method [18], the DNet [19], and the LCNN model [20]. The SEM method is one of the first data-driven local-filtering methods to use a deep learning based model for joint demosaicing and denoising. The DNet further improves the SEM model by adopting the convolutional neural network in its structure, while the LCNN model is a lightweight convolutional neural network to adopt residue learning and aggregated residual transformations.

These methods use a lot of images for the training of the network and use the conventional Bayer CFA. For the comparison of RGBW-CFA-based demosaicing, we compared with the demosaicing method developed by the Sony corporation [15] for the Sony RGBW CFA, with the Paul’s method [14] for the Paul’s RGBW CFA. We also compare with the residual interpolation method [6] as a representative of classic RGB demosaicing methods. We made quantitative comparisons in terms of the CPSNR (Color Peak Signal-to-Noise Ratio) the SSIM (Structural Similarity Index Measure), and the FSIMc (Feature Similarity Index for Color images).

**Figure 4:**
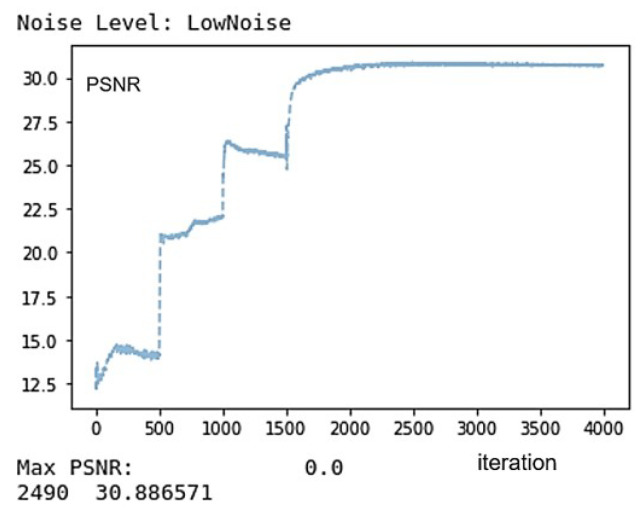
Showing the CPSNR values in the process of reconstructing the Kodak No. 17 image as the iteration progresses.

Figure 4 shows how the CPSNR value changes in the process of reconstructing the Kodak No. 17 image as the iteration progresses. It can be seen that after iteration 2100, the CPSNR value converges. Therefore, we normally terminate the reconstruction process at iteration 2100. Furthermore, it can be observed that the PSNR value changes significantly at iterations 500, 1000, and 1500 because the loss function changes at this time, as explained in Section 3.2.

**Figure 5:**
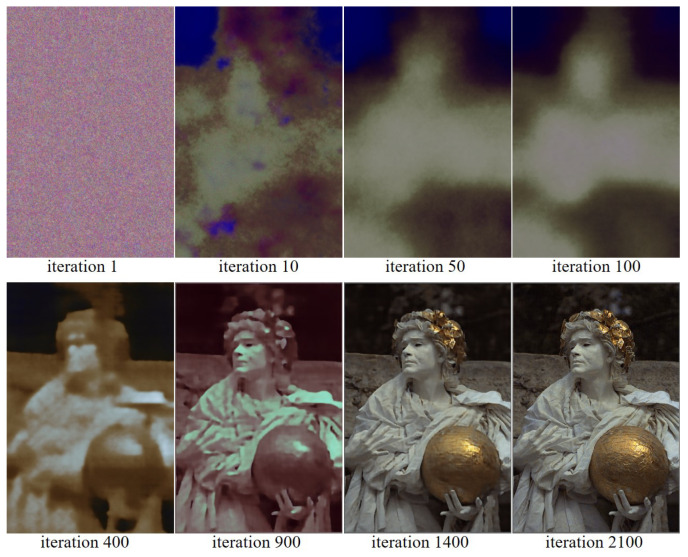
Showing the process of reconstructing the Kodak No. 17 image as the iteration progresses.

**Figure 6:**
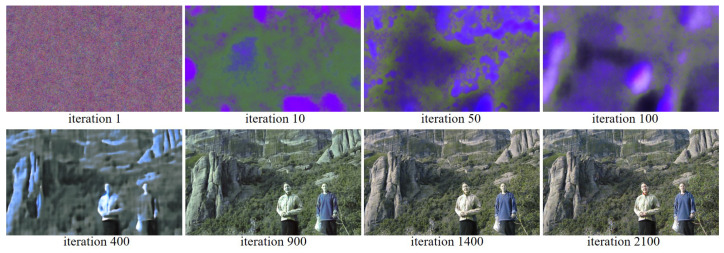
Showing the process of reconstructing the Kodak No. 25 image as the iteration progresses.

Figure 5 and Figure 6 show the reconstruction of the Kodak No. 17 and No. 25 images according to the progress of the iteration, respectively. Again, it can be observed that there is a large change in the colors, before and after 500, 1000, and 1500 iterations, respectively, as the loss function changes at iteration 500, 1000, and 1500, respectively. It can be seen that the all the colors are well reconstructed at iteration 2100.

**Figure 7:**
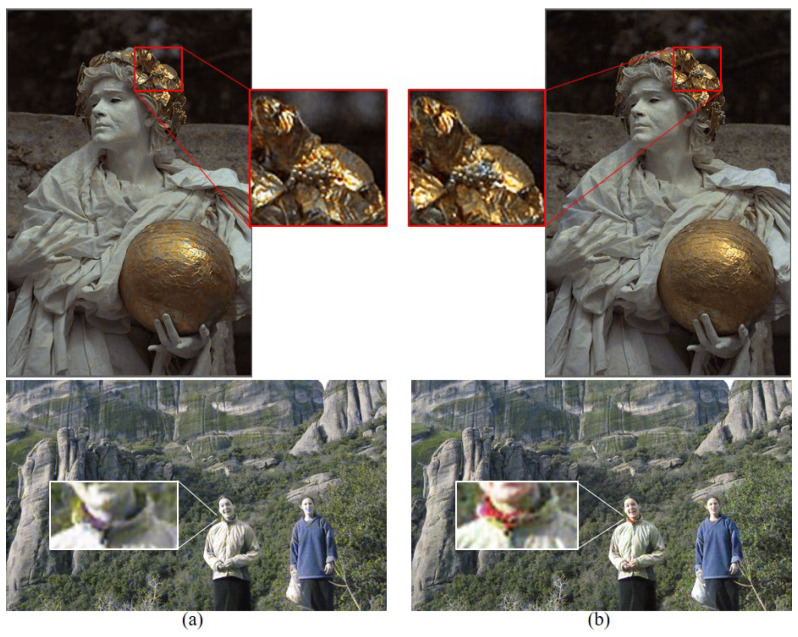
Comparing the reconstructions results with the proposed method on the random RGBW CFA on the Kodak No. 17 and No. 25 images with (**a**) a ratio of 1:2:1:4 of R, G, B, and W pixels (**b**) a ratio of 1:1:1:3 of R, G, B, and W pixels.

To show the effectiveness of using the same ratio R, G, and B pixels as opposed to the 1:2:1 ratio of R, G, and B pixels commonly used in conventional demosaicing methods, we compared, in Figure 3, the reconstruction results with the RGBW CFAs having a ratio 1:2:1:4 of R, G, B, W pixels, and a ratio of 1:1:1:3 of R, G, B, W pixels, respectively. To show the validity of using equal ratio R, G, and B pixels as opposed to the ratio of 1:2:1 of R, G, and B pixels normally used in conventional demosaicing methods, we compared in Figure 3 the reconstruction results with the proposed method on the random RGBW CFA on the Kodak No. 17 and No. 25 images with a ratio of 1:2:1:4 of R, G, B, W pixels, and a ratio of 1:1:1:3 of R, G, B, W pixels, respectively. Due to the larger proportion G pixels in the RGBW CFA with ratio of 1:2:1:4, the reconstructed images appear a bit greenish, as can be observed especially in the bottom image of Figure 7a. Furthermore, due to the smaller number of R and B pixels, some of the colors are not reconstructed, such as the red in the scarf in the bottom image of Figure 7a. However, with the proposed RGBW pattern, all the colors are well reconstructed.

**Figure 8:**
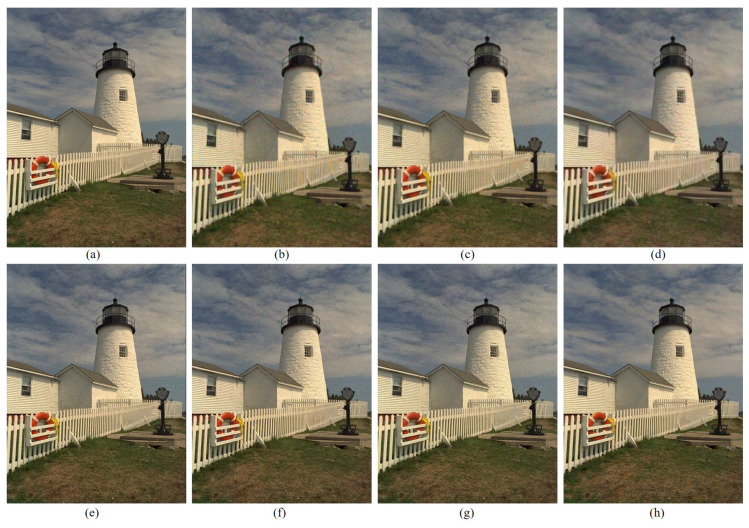
Visual comparison of the details between the results of different demosaicing methods on the Kodak No. 19 image: (**a**) original (**b**) RI [6] (**c**) Sony [15] (**d**) Paul’s [14] (**e**) DNet [19] (**f**) SEM [18] (**g**) LCNN [20] (**h**) Proposed.

**Figure 9:**
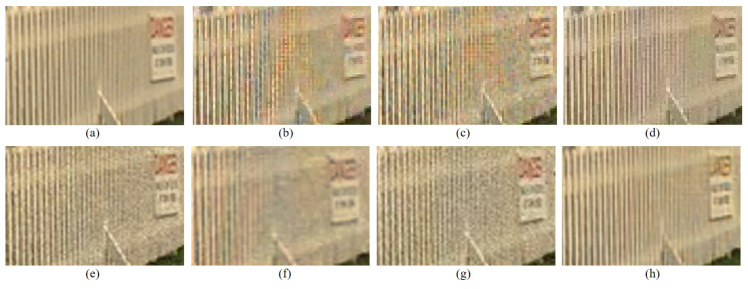
Visual comparison of the details in Figure 8 between the results of different demosaicing methods on the Kodak No. 19 image: (**a**) original (**b**) RI [6] (**c**) Sony [15] (**d**) Paul’s [14] (**e**) DNet [19] (**f**) SEM [18] (**g**) LCNN [20] (**h**) Proposed.

Table 1 summarizes the CPSNR, SSIM, and the FSIMc values of the various demosaicing methods, which are the average values for all the images in the Kodak and the McMaster datasets, respectively. The proposed method shows the highest results for all evaluation indicators. Figure 8 and Figure 9 show the visual comparison on the different methods on the Kodak No.19 image. The fence region in Kodak No.19 image is used a lot in the comparison between different demosaicing methods because it is difficult to reconstruct the fence bars while preventing the aliasing artifact. As can be observed in Figure 9b,c, the RI and the Sony CFAs with their corresponding demosaicking methods show aliasing artifacts (color artifacts) on the fence region. Paul’s method and SEM method can deal with aliasing artifacts to some extent but some aliasing artifacts still remain. The DNet and the LCNN methods can deal better with the aliasing artifacts but are not so efficient in eliminating the noise, as can be seen in Figure 9. Due to the smaller noise at white pixels, the effective reconstruction by the proposed loss function, and the inherent denoising property, the proposed method shows good denoising effects as can be seen in Figure 9h. However, one of the drawbacks of the proposed method is that the color fades a little, as can be seen in Figure 9h. There is still room for improvement if other network structures are used for the DIP, which may be one of the further study topics.

## 5. Conclusions

In this paper, we proposed a DIP-based joint demosaicing and denoising method tailored for the demosaicing of a white-dominant RGBW color filter array. The demosaicing is performed by training the DIP network with a single noisy color filter array (CFA) image. For this aim, we proposed a loss function different from those used in other DIP-based image restoration applications. We proposed how to reconstruct colors from white pixels that have no explicit color information and showed in the experimental results that the noise in the white pixels as well as in the R, G, and B pixels are well removed by the regression process inherent in the minimization of the loss function. The proposed method can easily be applied to the demosaicing to other color filter arrays.

## Figures and Tables

**Table 1:** Comparison of the CPSNR, SSIM, and the FSIMc values between the various demosaicing methods on the Kodak and the McMaster image datasets with Low Noise Level. The largest values are in bold fonts.

Dataset	Method	CPSNR	SSIM	FSIMc
	SEM [18]	30.39	0.9961	0.9792
	DNet [19]	28.79	0.9540	0.9743
	LCNN [20]	29.00	0.9948	0.9932
Kodak	RI [6]	28.69	0.9517	0.9448
	Sony [15]	29.17	0.9615	0.9502
	Paul et al. [14]	30.72	0.9794	0.9566
	Proposed	**30.80**	**0.9964**	**0.9953**
	SEM [18]	29.26	0.9940	0.9773
	DNet [19]	28.88	0.9579	0.9756
	LCNN [20]	28.94	0.9943	0.9922
McMaster	RI [6]	28.59	0.9563	0.9456
	Sony [15]	28.80	0.9641	0.9515
	Paul et al. [14]	27.79	0.9715	0.9571
	Proposed	**31.61**	**0.9963**	**0.9936**
